# Supplementary figures and images for: Malian children infected with Plasmodium ovale and Plasmodium falciparum display very similar gene expression profiles
Source: PLoS Negl Trop Dis. 2023 Jan 25;17(1):e0010802. doi: 10.1371/journal.pntd.0010802 (PMC9901758; doi:10.1371/journal.pntd.0010802)

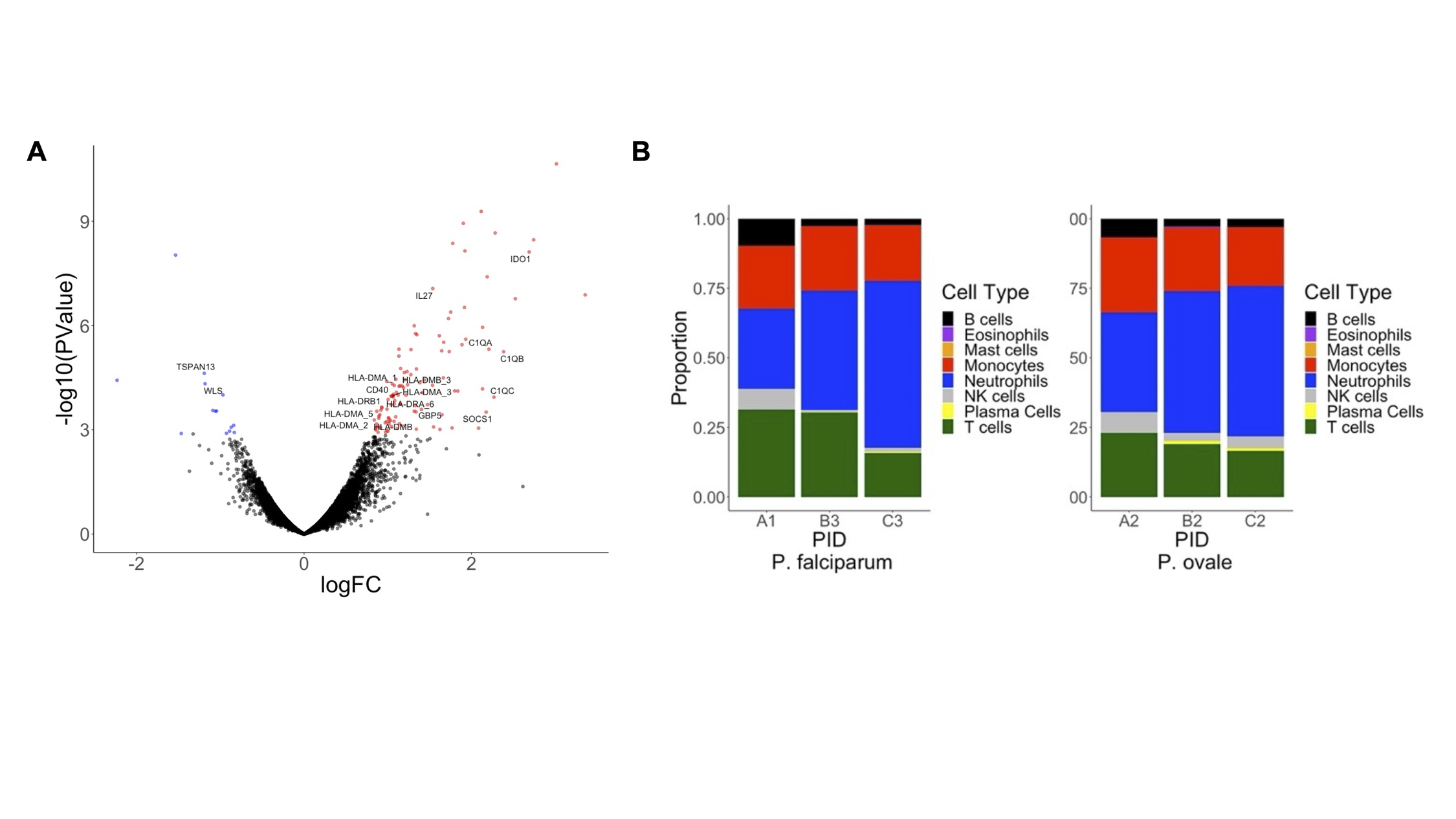

Supplement: S1 Fig — (A) Differences in host gene expression between P. ovale and P. falciparum infections. Each dot represents a human gene and is displayed according to the log fold-change (x-axis) and -log10 p-value (y-axis) and colored according to the statistical significance (black–non-significant, red–significantly overexpressed in P. falciparum infections, blue–significantly overexpressed in P. ovale infections, FDR = 0.1). (B) Gene expression deconvolution results of infections with P. falciparum (left) or P. ovale (right). Chi-square tests were performed for each individual to compare the immune cell composition during P. falciparum and P. ovale infections. Individual A: X2 = 2.99, p = 0.56, Individual B: X2 = 7.77, p = 0.10, Individual C: X2 = 0.89, p = 0.93 (TIF) [file pntd.0010802.s001.tif]

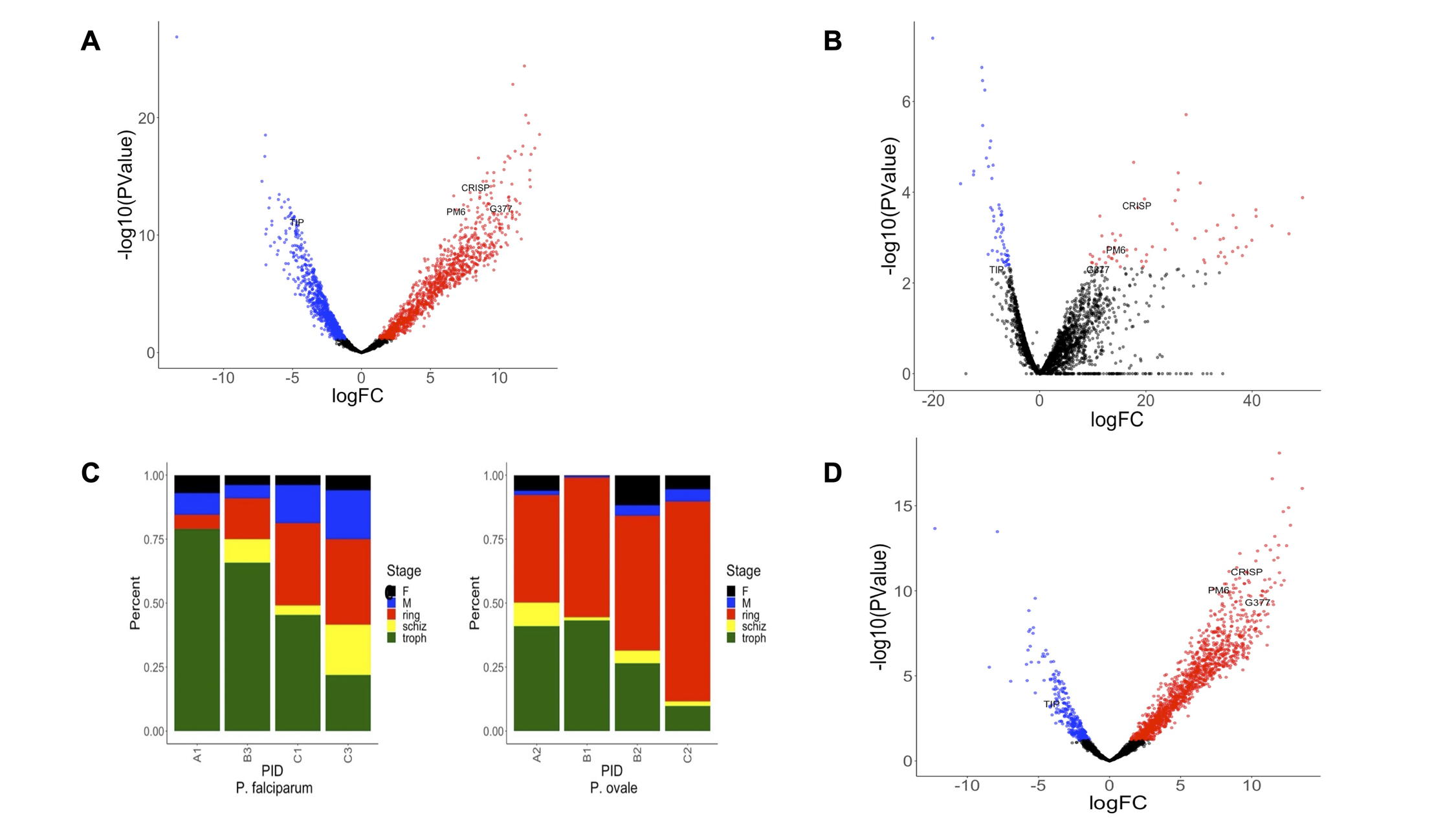

Supplement: S2 Fig — (A, B) Differences in parasite gene expression between P. ovale and. P. falciparum infections. Each dot represents a parasite gene and is displayed according to the log fold-change (x-axis) and -log10 p-value and colored according to the statistical significance (black–non-significant, red–significantly overexpressed in P. falciparum infections, blue–significantly overexpressed in P. ovale infections, FDR = 0.1). The volcano plots show the results without correcting the analyses for stage composition differences (A) or after correction (B). (C) Gene expression deconvolution results from Plasmodium RNA-seq reads during infection with P. falciparum (left) or P. ovale (right). (TIF) [file pntd.0010802.s002.tif]

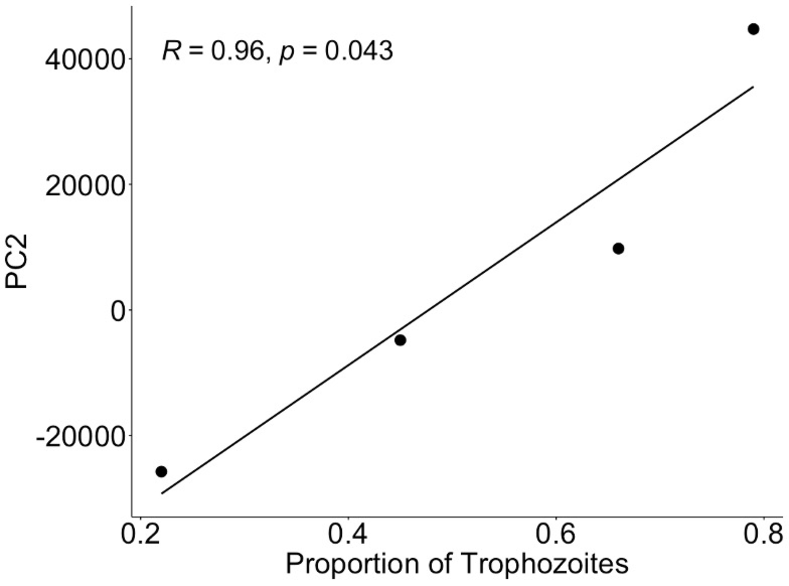

Supplement: S3 Fig — The scatterplot shows the estimated proportion of trophozoites present in each P. falciparum sample (x-axis) relative to the position of this infection along PC2 of Fig 2 (y-axis). (TIF) [file pntd.0010802.s003.tif]

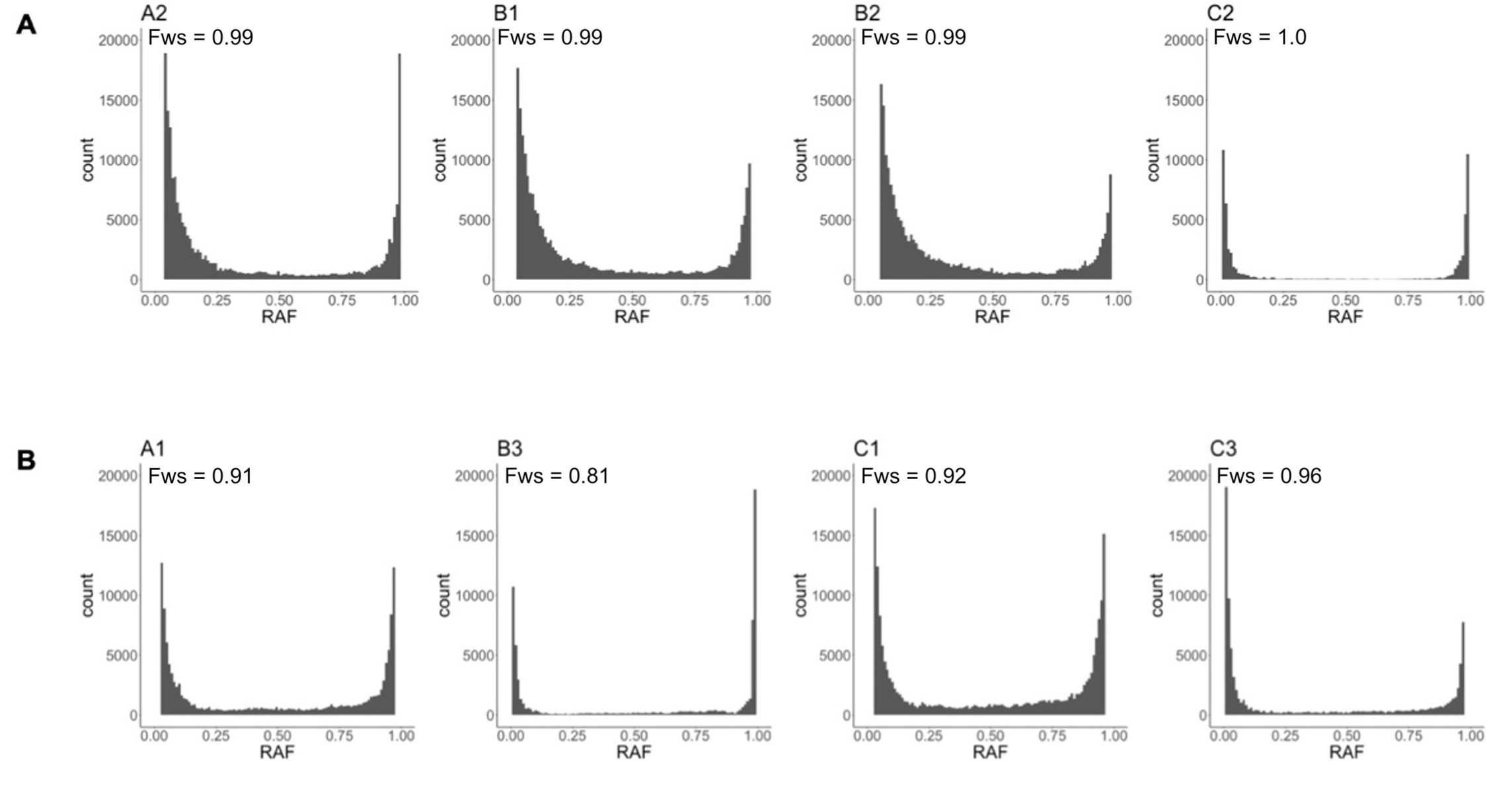

Supplement: S4 Fig — Complexity of the P. falciparum (A) and P. ovale (B) infections. Each plot shows the number of nucleotide positions (y-axis) with a particular reference allele frequency (x-axis, from 0 –all reads supporting an alternative allele, to 100%—all reads supporting the reference sequence allele). Note the U-shape distributions indicating the monoclonality of the infections. (TIF) [file pntd.0010802.s004.tif]

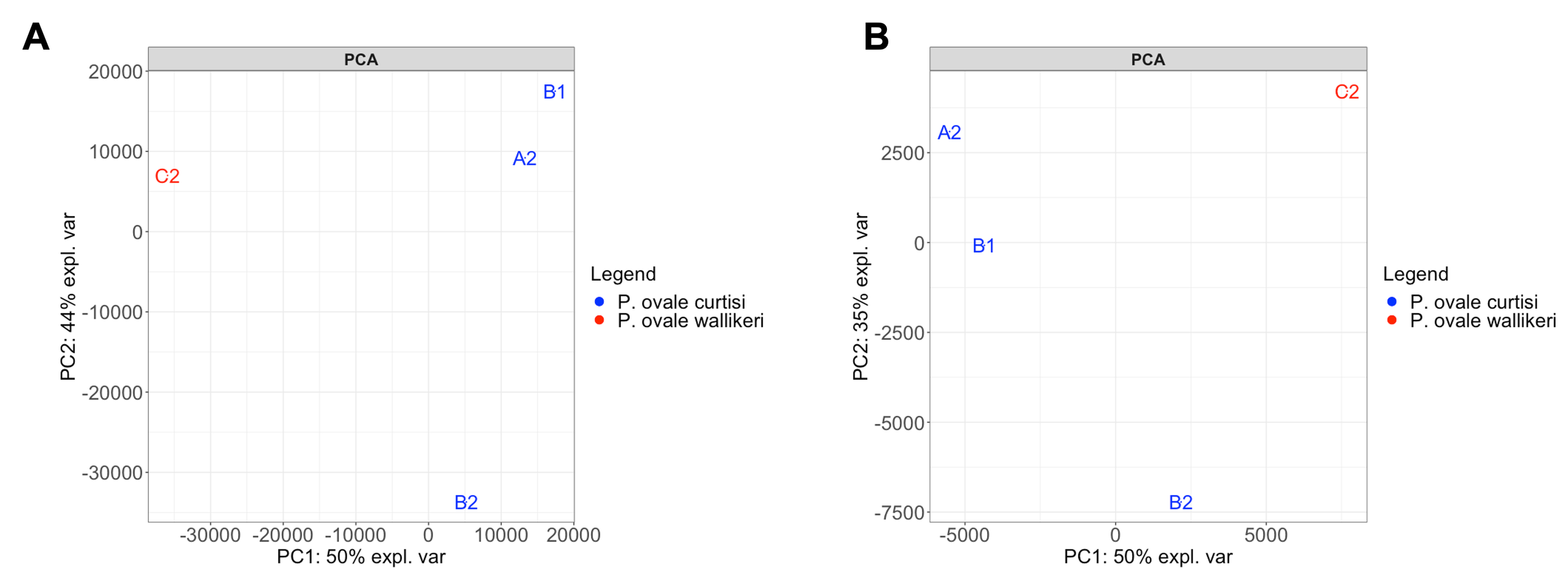

Supplement: S5 Fig — (A) PCA of human gene expression during infection. (B) PCA of parasite gene expression during infection. (TIF) [file pntd.0010802.s005.tif]
