# Supplementary material for: Malian children infected with Plasmodium ovale and Plasmodium falciparum display very similar gene expression profiles
Source: PLoS Negl Trop Dis. 2023 Jan 25;17(1):e0010802. doi: 10.1371/journal.pntd.0010802 (PMC9901758; doi:10.1371/journal.pntd.0010802)
Supplement: Attachment — Submitted filename: PNTD-D-22-01170_reviewer_CMM.pdf [file pntd.0010802.s010.pdf]

PLOS Neglected Tropical Diseases

Malian children infected with Plasmodium ovale and Plasmodium falciparum display very similar gene expression profiles.

--Manuscript Draft--

Manuscript Number:	PNTD-D-22-01170
Full Title:	Malian children infected with Plasmodium ovale and Plasmodium falciparum display very similar gene expression profiles.
Short Title:	Gene expression profiles of Malian children infected with P. falciparum or P. ovale
Article Type:	Research Article
Keywords:	plasmodium; gene expression; plasmodium ovale; plasmodium falciparum; RNA-seq; dual RNA-seq
Abstract:	Plasmodium parasites caused 241 million cases of malaria and over 600,000 deaths in 2020. Both P. falciparum and P. ovale are endemic to Mali and cause malaria illness, with P. falciparum infections typically being more severe. Here, we sequenced RNA from nine pediatric blood samples collected during uncomplicated, symptomatic infections with either P. falciparum or P. ovale and characterized the host and parasite gene expression profiles. We found that human gene expression varies more between individuals than according to the parasite species causing the infection, while parasite gene expression profiles cluster by species. Additionally, we characterized DNA polymorphisms of the parasites directly from the RNA-seq reads and found comparable levels of genetic diversity in both species despite dramatic differences in prevalence. Our results provide unique insights into host-pathogen interactions during malaria infections and their variations according to the infecting Plasmodium species, which will be critical to develop better elimination strategies against all human Plasmodium parasites.
Additional Information:	
**Question**	**Response**
**Financial Disclosure**<br><br>Enter a financial disclosure statement that describes the sources of funding for the work included in this submission. Review the submission guidelines for detailed requirements. View published research articles from *PLOS NTDs* for specific examples.<br><br>This statement is required for submission and **will appear in the published article** if the submission is accepted. Please make sure it is accurate.	This work was supported by awards from the National Institutes of Health (R21AI146853 and R01HL146377) and an NIAID-funded predoctoral fellowship (T32 AI095190). Participant enrollment and sample collection were supported by NIH grants U01AI065683, R01HL130750, R01AI099628, and D43TW001589. The funders had no role in study design, data collection and analysis, decision to publish, or preparation of the manuscript.

Powered by Editorial Manager® and ProduXion Manager® from Aries Systems Corporation

**Unfunded studies**<br><br>Enter: *The author(s) received no specific funding for this work.*<br><br>**Funded studies**<br><br>Enter a statement with the following details:<br>• Initials of the authors who received each award<br>• Grant numbers awarded to each author<br>• The full name of each funder<br>• URL of each funder website<br>• Did the sponsors or funders play any role in the study design, data collection and analysis, decision to publish, or preparation of the manuscript?<br>• **NO** - Include this sentence at the end of your statement: *The funders had no role in study design, data collection and analysis, decision to publish, or preparation of the manuscript.*<br>• **YES** - Specify the role(s) played.<br><br>* typeset	
**Competing Interests**<br><br>Use the instructions below to enter a competing interest statement for this submission. On behalf of all authors, disclose any competing interests that could be perceived to bias this work—acknowledging all financial support and any other relevant financial or non-financial competing interests.<br><br>This statement **will appear in the published article** if the submission is accepted. Please make sure it is accurate. View published research articles from *PLOS NTDs* for specific examples.	The authors have declared that no competing interests exist.

**NO authors have competing interests**<br><br>Enter: *The authors have declared that no competing interests exist.*<br><br>**Authors with competing interests**<br><br>Enter competing interest details beginning with this statement:<br><br>*I have read the journal's policy and the authors of this manuscript have the following competing interests: [insert competing interests here]*<br><br>* typeset<br><br>This statement is required for submission and **will appear in the published article** if the submission is accepted. Please make sure it is accurate and that any funding sources listed in your Funding Information later in the submission form are also declared in your Financial Disclosure statement.	
**Data Availability**<br><br>Authors are required to make all data underlying the findings described fully available, without restriction, and from the time of publication. PLOS allows rare exceptions to address legal and ethical concerns. See the PLOS Data Policy and FAQ for detailed information.	Yes - all data are fully available without restriction

A Data Availability Statement describing where the data can be found is required at submission. Your answers to this question constitute the Data Availability Statement and **will be published in the article**, if accepted.<br><br>**Important:** Stating 'data available on request from the author' is not sufficient. If your data are only available upon request, select 'No' for the first question and explain your exceptional situation in the text box.<br><br>Do the authors confirm that all data underlying the findings described in their manuscript are fully available without restriction?	
**Describe where the data may be found in full sentences. If you are copying our sample text, replace any instances of XXX with the appropriate details.**<br><br>• If the data are **held or will be held in a public repository**, include URLs, accession numbers or DOIs. If this information will only be available after acceptance, indicate this by ticking the box below. For example: *All XXX files are available from the XXX database (accession number(s) XXX, XXX.)*.<br>• If the data are all contained **within the manuscript and/or Supporting Information files**, enter the following: *All relevant data are within the manuscript and its Supporting Information files.*<br>• If neither of these applies but you are able to provide **details of access elsewhere**, with or without limitations, please do so. For example:<br><br>*Data cannot be shared publicly because*	All sequence files are available in the Sequence Read Archive under the BioProject PRJNA878485.

of [XXX]. Data are available from the XXX Institutional Data Access / Ethics Committee (contact via XXX) for researchers who meet the criteria for access to confidential data.<br><br>*The data underlying the results presented in the study are available from (include the name of the third party and contact information or URL).*<br>• This text is appropriate if the data are owned by a third party and authors do not have permission to share the data.<br><br>* typeset	
Additional data availability information:	Tick here if the URLs/accession numbers/DOIs will be available only after acceptance of the manuscript for publication so that we can ensure their inclusion before publication.

Powered by Editorial Manager® and ProduXion Manager® from Aries Systems Corporation

manuscript text
Click here to access/download;Manuscript;PoPf_RNAseq_maintext.docx

Malian children infected with *Plasmodium ovale* and *Plasmodium falciparum* display very similar gene expression profiles.

Kieran Tebben[1,2], Salif Yirampo[3], Drissa Coulibaly[3], Abdoulaye K. Koné[3], Matthew B. Laurens[4], Emily M. Stucke[4], Ahmadou Dembélé[3], Youssouf Tolo[3], Karim Traoré[3], Amadou Niangaly[3], Andrea A. Berry[4], Bourema Kouriba[3], Christopher V. Plowe[4], Ogobara K Doumbo[3†], Kirsten E. Lyke[4], Shannon Takala-Harrison[4], Mahamadou A. Thera[3], Mark A. Travassos[4], and David Serre[1,2*]

[1]Institute for Genome Sciences, University of Maryland School of Medicine, Baltimore, MD, USA

[2]Department of Microbiology and Immunology, University of Maryland School of Medicine, Baltimore MD 21201, USA

[3]Malaria Research and Training Center, University of Sciences, Techniques and Technologies, Bamako, Mali

[4]Malaria Research Program, Center for Vaccine Development and Global Health, University of Maryland School of Medicine, Baltimore, MD, United States of America

[†]Deceased

*Correspondence to dserre@som.umaryland.edu

Abstract

Plasmodium parasites caused 241 million cases of malaria and over 600,000 deaths in 2020. Both *P. falciparum* and *P. ovale* are endemic to Mali and cause malaria illness, with *P. falciparum* infections typically being more severe. Here, we sequenced RNA from nine pediatric blood samples collected during uncomplicated, symptomatic infections with either *P. falciparum* or *P. ovale* and characterized the host and parasite gene expression profiles. We found that human gene expression varies more between individuals than according to the parasite species causing the infection, while parasite gene expression profiles cluster by species. Additionally, we characterized DNA polymorphisms of the parasites directly from the RNA-seq reads and found comparable levels of genetic diversity in both species despite dramatic differences in prevalence. Our results provide unique insights into host-pathogen interactions during malaria infections and their variations according to the infecting *Plasmodium* species, which will be critical to develop better elimination strategies against all human *Plasmodium* parasites.

Author Summary

Multiple species of *Plasmodium* parasites can cause human malaria. Most studies and elimination efforts target *P. falciparum,* the most common cause of malaria worldwide and the species responsible for the vast majority of the mortality. Other *Plasmodium* species, such as *P. ovale,* typically lead to less severe forms of the disease but little is known about the molecular mechanisms at play during malaria infections with different parasites. We analyzed host and parasite gene expression from children successively infected with *P. ovale* and *P. falciparum* and found that, while the parasite gene expression differed significantly, the transcriptional profiles of the host immune cells were similar in *P. ovale*

or *P. falciparum* infections. This suggests that infected individuals respond to uncomplicated malaria infections similarly, regardless of the *Plasmodium* species causing the infection, and that alternative immune processes may become important during the progression to severe *P. falciparum* malaria (rather than being inherent features of *P. falciparum* infections). Additionally, we observed similar levels of genetic diversity among *P. ovale* and *P. falciparum* parasites, suggesting that the *P. ovale* population might be larger than currently thought, possibly due to extensive misdiagnosis or the existence of hidden reservoirs of parasites.

Introduction

Plasmodium parasites caused 241 million cases of malaria and over 600,000 deaths in 2020(1), a partial reversal of decades of progress towards elimination. Malaria symptoms derive from the asexual replication of *Plasmodium* parasites in human red blood cells (RBCs)(2). At least five species of *Plasmodium* parasites commonly cause human malaria: *P. falciparum, P. vivax, P. ovale, P. malariae* and *P. knowlesi*(2). *P. falciparum* is responsible for the majority of infections worldwide and is the dominant species in Sub-Saharan Africa(2,3). *P. falciparum* can cause severe malaria(2) and is responsible for the majority of malaria deaths through complications such as severe anemia or cerebral malaria. This high pathogenicity is thought to be, at least partially, due to the sequestration of mature asexual *P. falciparum* parasites in the microvasculature(4). Post-mortem analyses of brain(5) or kidney(6) from individuals with severe malaria show parasite accumulation in the tissue microvasculature. This accumulation can lead to obstructions and focal hypoxia, and local increase in inflammatory cells and molecules responding to

the parasites(5). Rupture of adhered infected RBCs after schizont maturation can also lead to focal release of parasite antigens and immune-activating factors, contributing to localized tissue damage at the site of adherence(6). While most studies focus on *P. falciparum*, other *Plasmodium* species also cause significant public health issues and our lack of specific knowledge about these parasites is becoming increasingly problematic. *P. vivax* is common in South Asia and South America but rare in Sub-Saharan Africa(1,7), while *P. knowlesi* is a recent zoonotic parasite causing infections in Southeast Asia(8). *P. ovale* and *P. malariae* are widely distributed parasites that have typically been considered as relatively rare(9) and causing milder infections than *P. falciparum*(3,10,11). Infection with these parasites typically leads to lower fevers(10) and parasitemia(10,11) than those with *P. falciparum,* possibly due to a slower intraerythrocytic replication with fewer merozoites produced per cycle(11) and a preference for specific RBCs(10,11). Because these parasites are difficult to detect on peripheral blood smear(10,11) and often occur in coinfections with more virulent species(9), they are likely underdiagnosed(9). *P. ovale* is further categorized into two phenotypically indistinguishable sub-species or species(12), *P. ovale curtisi* and *P. ovale wallikeri*, that are often co-endemic, notably in West Africa(2,3,13).

Since blood-stage parasites play a central role in disease severity, transmission and immunity/immune-evasion, analyses of host and parasite gene expression from infected blood samples could provide critical information on these processes and their regulation. Such studies have been performed from *P. falciparum* infections, either separately to examine changes in host(14–16) or parasite(17–22) transcriptional regulation during infections, or jointly(23–26) from the same samples to investigate host-pathogen

interactions. By contrast, a single study has characterized the gene expression profiles of *P. ovale* parasites during an infection(27), and none have examined the host transcriptome during infections with this parasite.

Here, we used dual RNA-sequencing (dual RNA-seq) to examine whole blood samples from three Malian children successively infected with *P. falciparum* and *P. ovale,* and simultaneously characterize the gene expression profiles of the host and parasites. Our analyses provide novel transcriptomic and genetic insights on *P. ovale* infections and allow a first examination of the host immune response to these infections and how this response differs from the immune response to *P. falciparum* infections.

Results

Characterization of host and parasite gene expression profiles by dual RNA-seq

We extracted and sequenced RNA from nine blood samples collected from three Malian children successively infected with *P. falciparum* and *P. ovale* (**Table 1**). To confirm the *Plasmodium* species responsible for the symptomatic malaria episodes, we simultaneously mapped all reads to the genomes of *P. falciparum*, *P. malariae*, *P. ovale* and *P. vivax*. In each sample, more than 98% of the *Plasmodium* reads mapped to the species identified by thick smear microscopy with the exception of one sample (C2) for which 93.9% of the *Plasmodium* reads mapped to *P. ovale* (as determined by microscopy) and 5.4% of the reads mapped to *P. falciparum*, possibly suggesting a co-infection (**Supplemental Table 1**).

Table 1: Epidemiological and clinical characteristics of study participants.

Sample	Sex	Ethnicity	Age	Infecting parasite species	Parasitemia (parasites/µL blood)	Temperature (ºC)	Hemoglobin conc. (g/dL)
A1	Female	Dogon	5	*P. falciparum*	16,950	36	8.9
A2			7	*P. ovale*	2,025	38.1	8.9
B1	Female	Bambara	9	*P. ovale*	11,375	37.8	12.4
B2			9	*P. ovale*	800	39.5	11.5
B3			8	*P. falciparum*	600	37.2	11.9
B4			8	*P. falciparum*	18,300	37.4	12
C1	Male	Dogon	12	*P. falciparum*	131,700	38	10.9
C2			13	*P. ovale*	4,825	38.9	10.9
C3			13	*P. falciparum*	11,950	38.2	11

To analyze the gene expression profiles of the host and parasites in each infection, we then mapped all reads simultaneously to the *P. falciparum*, *P. ovale* and human genomes (**Supplemental Table 2**). Overall, for each sample, we obtained more than 48 million reads (70-92%) mapped to the human genome and more than 1 million reads (2-25%) mapped to the *Plasmodium* genome, providing robust characterization of the host and parasite gene expression profiles of each infection.

Host gene expression varies more between individuals than between infecting species

We first used principal component analysis (PCA) to examine the relationships among the host gene expression profiles characterized from each *P. falciparum* and *P. ovale* infection. Interestingly, the host gene expression profiles seemed to cluster according to the participant rather than by the infecting parasite species **(Figure 1A)**. To rigorously quantify this observation, we calculated the proportion of the variance in gene expression(28) explained by the infecting parasite species, the parasitemia, the age of the participant at the time of the infection, and inter-individual differences **(Figure 1B)**.

Consistent with the PCA, the infecting parasite species explained less than 5% of the variance in host gene expression (median: 0%, IQR: 0% – 2.2%). By contrast, the child's age at the time of infection explained, on average, a third of the variance (median: 34%, IQR: 11.1% – 57%). Note that given the small number of samples analyzed here, it was difficult to rigorously determine the variance in gene expression explained by inter-individual differences vs. age (or sex and ethnicity that cannot be considered here) as these variables are confounded. However, these analyses clearly showed that the variance in host gene expression during a *Plasmodium* infection was primarily driven by host factors and that the infecting parasite species had, comparatively, little effect.

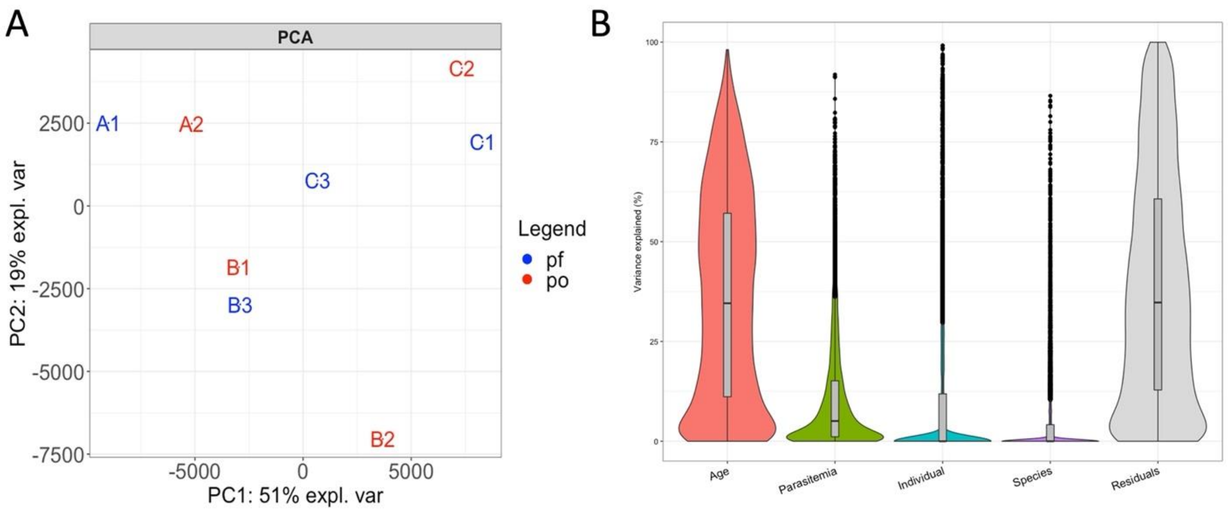

Figure 1: Host gene expression differs more between individuals than according to the infecting species. (**A**) PCA showing the relationships among the nine infections based on the expression level of 9,884 human genes and colored according to the infecting species (blue – *P. falciparum*, red – *P. ovale*). (**B**) Percentage of the variance in host gene expression explained by the age of the child, the parasitemia of the infection, the individual and the infecting species.

The few host genes differentially expressed between P. falciparum and P. ovale infections are involved in adaptive immunity

We then tested whether specific host genes were differentially expressed in *P. ovale vs. P. falciparum infections.* To account for the important effects of inter-individual differences on host gene expression, we used a paired design for these statistical analyses: we selected two sequential samples per individual, one infected with *P. falciparum* and one infected with *P. ovale,* to minimize differences in the age of the participant and other inter-individual factors. Overall, out of 9,884 genes tested, we only identified 127 human genes that were differentially expressed between *P. ovale* and *P. falciparum* infections (FDR<0.1, **Supplemental Figure 1A, Supplemental Table 3**), confirming their overall similarities. To understand whether these differences in gene expression were a result of i) true differences in gene regulation or ii) differences in immune cell composition, we used CIBERSORTx(29) to estimate, directly from the RNA-seq reads, the relative proportion of each immune cell subset present in each *P. falciparum*- and *P. ovale* infected sample **(Supplemental Figure 1B)**. We found no significant differences in immune cell composition between the *P. falciparum* and *P. ovale* infections in any of the three individuals (chi-squared test, $p > 0.1$), suggesting that differentially expressed host genes reflect true changes in expression rather than differences in white blood cell composition. This overall similarity in relative proportions of immune cell populations during *P. ovale* and *P. falciparum* infections might be surprising given the differences in disease severity caused by each species(3). However, it is important to note here that all infections included were uncomplicated symptomatic infections with relatively similar presentation (i.e., temperature, hemoglobin level and clinical assessment, **Table 1**),

which suggests that alternative immune processes and immune cell recruitment may become important during the progression to severe *P. falciparum* malaria rather than being inherent features of *P. falciparum* infections.

Among the host genes significantly upregulated in *P. falciparum* infections, compared to *P. ovale* infections, we observed genes involved in the suppression of T-cell mediated responses to pathogens, such as *ido1*(30,31) and *il27*(32–34). By contrast, several genes related to dendritic cell development (a particularly important cell type for bridging the innate and adaptive immune responses) were overexpressed in *P. ovale* compared to *P. falciparum* infections **(Supplemental Figure 1A)**. For example, *wls* has been reported to be essential for dendritic cell homestasis(35) and *tspan13* is highly expressed in plasmacytoid dendritic cells(36). Overall, these findings possibly suggest a stronger suppression of the adaptive immune response (particularly T-cells) during *P. falciparum* than *P. ovale* infections and a stronger activation of the adaptive immune response during *P. ovale* infections by promoting dendritic cell development.

Plasmodium gene expression differs more by species than by infected host

RNA-seq data generated directly from infected blood samples enables simultaneous characterization of host and parasite gene expression and we next focused on examining differences in parasite gene expression. To compare the gene expression of *P. falciparum* and *P. ovale* parasites during symptomatic infections, we only included 2,631 expressed genes that had one-to-one orthologs in both species (see Material and Methods). Again, we first used PCA to investigate how global gene expression differs between uncomplicated symptomatic infections with *P. falciparum* and *P. ovale.* In contrast to host

gene expression, the parasite gene expression profiles from each species were clearly separated by PC1 **(Figure 2A)**, with *P. ovale* infections clustering closely together and the *P. falciparum* samples spread along PC2. Indeed, on average one third of the variance in parasite gene expression was explained by the parasite species (median: 39.1%, IQR: 10.8% - 61.6%)**,** while the age of the child explained 14.6% of the variance, and the parasitemia and individual differences less than 6%, on average **(Figure 2B)**. While not surprising, these findings are in stark contrast with the patterns observed for the host gene expression profiles of the same infections, suggesting that while inducing a relatively similar response of the host, *P. falciparum* and *P. ovale* parasites are regulated differently in the blood.

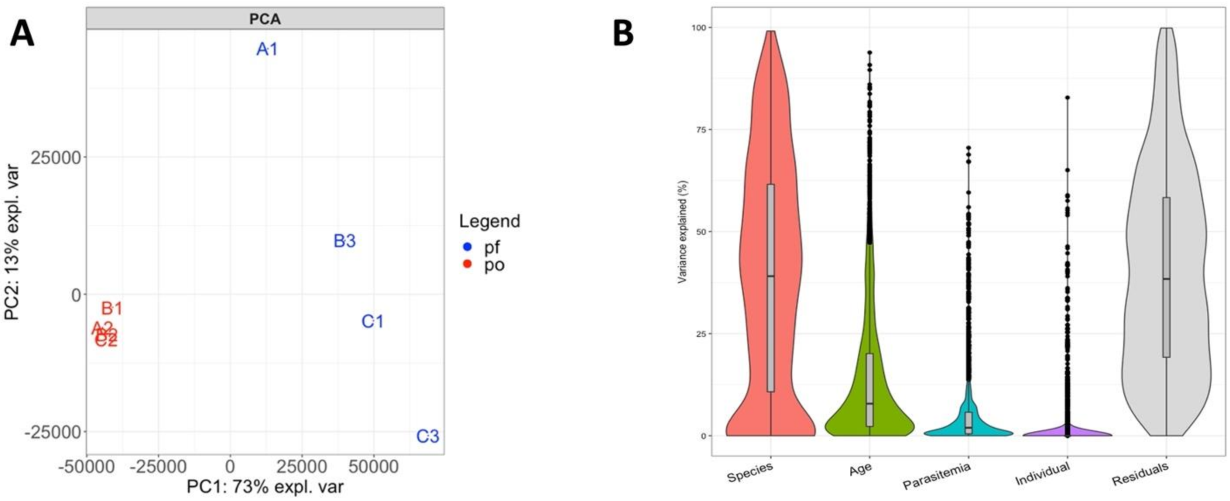

Figure 2: *Plasmodium* gene expression profiles during symptomatic infections cluster according to the infecting species. **(A)** PCA showing the relationships among the nine infections based on the expression level of 2,631 *Plasmodium* genes and colored according to the infecting species (blue – *P. falciparum*, red – *P. ovale*). (**B)** Percentage of the variance in parasite gene expression explained by the infecting species, the age of the child, the parasitemia of the infection and the individual.

Differences in parasite gene expression in P. ovale and. P. falciparum infections are largely explained by differences in stage composition

We then statistically tested which orthologous parasites genes were differentially expressed in *P. falciparum* vs. *P. ovale* infections, and since host factors (i.e., immunity, age, sex) seemed to contribute little to the variations in parasite gene expression, we included all samples from both species in our statistical analyses.

We identified 1,858 parasite genes that were differentially expressed according to the parasite species (FDR < 0.1, **Supplemental Figure 2A**, **Supplemental Table 4**). To understand whether these differences were the result of i) true differences in gene expression or ii) differences in the relative proportion of the various parasite developmental stages present in each blood sample, we used a species-agnostic gene expression deconvolution(37) to estimate the stage composition of each *P. falciparum* and *P. ovale* sample **(Supplemental Figure 2C)**. The stage compositions of *P. falciparum* infections appeared more variable than those of *P. ovale* infections and, in fact, the relative proportion of trophozoites in *P. falciparum* infections was correlated with the overall variation in gene expression captured by PC2 (p = 0.043) **(Supplemental Figure 3)**. This observation is surprising since one would expect *P. falciparum* ring stage parasites to be highly predominant in peripheral blood (since mature *P. falciparum* blood stages usually sequester in tissues(4)) while sequestration has not been described for *P. ovale*(10). Note that the stage composition estimated by gene expression deconvolution reflects the proportions of transcripts derived from each stage and, since rings are less

transcriptionally active than other blood stages(20), the proportion of ring stage parasites is systematically (but proportionally) underestimated.

Accounting for stage composition differences, only 118 orthologous genes remained differentially expressed between parasite species **(Supplemental Figure 2B**, **Supplemental Table 4)**, confirming that stage composition differences explained the vast majority of parasite gene expression differences observed between *P. ovale* and *P. falciparum* infections. A few genes associated with gametocyte function seemed to be significantly more expressed in *P. ovale* vs. *P. falciparum* infections (e.g., *g377*(38), *crisp*(39), and *pm6*(40)), possibly suggesting differential regulation of the parasite sexual stages between species. Conversely, a putative homolog of T-cell immunomodulatory protein (*tip*(41)), which has been described to have anti-inflammatory effects in *P. berghei* models, was expressed at higher levels in *P. falciparum* infections, consistent with the observation of T-cell suppression in human data and suggesting that a parasite factor was potentially associated with this process. However, with only nine infections examined, our gene expression analyses should be interpreted with caution and larger sample sizes will be required to confirm these findings.

RNA-seq data provide a preliminary assessment of the genetic diversity of P. falciparum and P. ovale symptomatic infections in Mali

Since RNA-seq provides a characterization of the gene expression by sequencing mRNA molecules, one can leverage these data to examine genetic variants located in the expressed transcripts. Between 3,027,680 and 10,274,779 nucleotide positions of the

Plasmodium genome were sequenced at >20X in each sample, allowing a robust investigation of the parasite genetic diversity (**Supplemental Table 2**).

We first examined whether we could detect allelic variations within each sample, which would indicate the presence of multiple genetically different parasites in the circulation. At all nucleotide positions sequenced, we only observed one allele **(Supplemental Figure 4)** demonstrating that all infections examined here were monoclonal.

We then focused on genetic differences between parasites from different infections. Since two sub-species of *P. ovale* can cause human malaria(12), we reconstructed, directly from the RNA-seq data, the entire cytochrome B gene sequence from each *P. ovale* infection and compared them with published sequences for both *P. ovale curtisi* and *P. ovale wallikeri*. The sequences generated from the infections A2, B1 and B2 clearly clustered with *P. ovale curtisi* sequences, while the sequence reconstructed from infection C2 clustered with *P. ovale wallikeri* sequences **(Figure 3A)**. Interestingly, despite the ancient divergence of the two *P. ovale* sub-species(9),(12), the parasite **(Figure 2)** or host **(Figure 1)** gene expression profiles generated from infections with these parasites were undistinguishable when analyzed with *P. falciparum* infections, suggesting that both subspecies were similarly regulated in blood infections and had, overall, similar consequences on host gene expression. (Note that when only *P. ovale* infections were considered, the gene expression profiles derived from the *P. ovale wallikeri* infection seemed to be distinct from those generated from *P. ovale curtisi* (**Supplemental Figure 5**) although the small number of samples analyzed prevented any definitive conclusion.

We then examined genome-scale differences in genetic diversity among the *P. falciparum* and *P. ovale* isolates by calculating the proportion of pairwise nucleotide differences

between each pair of samples of the same species. While both *Plasmodium* species are endemic in Mali(42), *P. falciparum* is much more prevalent than *P. ovale*(43) and one might therefore have expected a higher diversity among *P. falciparum* parasites. However, on average the proportion of differences was very similar between *P. falciparum* (mean: 0.00035, sd: 0.00013) and *P. ovale curtisi* (mean: 0.00028, sd: 0.00011) (t-test p-value = 0.44) isolates **(Figure 3B)** suggesting that despite their likely smaller population size, *P. ovale* parasites maintain a high genetic diversity.

A

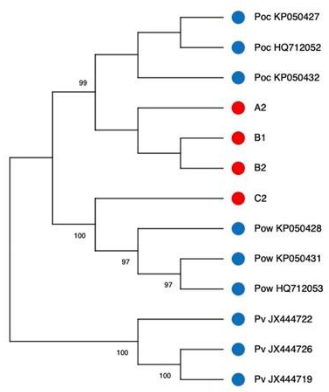

B

P. falciparum

	A1	B4	B3	C1
B4	0.00031			
B3	0.00032	0.00011		
C1	0.00051	0.00036	0.00030	
C3	0.00050	0.00025	0.00031	0.00053

P. ovale

	A2	B1	B2
B1	0.00034		
B2	0.00016	0.00035	
C2	0.011	0.012	0.011

Figure 3: Genetic relationships among the *Plasmodium* parasites analyzed by RNA-seq. **(A)** Neighbor-joining tree showing the relationships among cytochrome B sequences from the *P. ovale* parasites studied in this study (red) and sequences from NCBI (blue). Note that the sequences from the A2, B1 and B2 infections cluster with *P. ovale curtsi* sequences while the sequence from infection C2 clusters with *P. ovale wallikeri* sequences. The number next to each branch indicates the percentage of replicate trees in which the sequences clustered together based on 500 bootstraps. **(B)** Proportion of pairwise nucleotide differences between pairs of *P. falciparum* (top) and *P. ovale* (bottom) infections based on positions covered at >20X. Note the higher proportion of pairwise nucleotide differences in pairs including the C2 infection, consistent with its *P. ovale*

wallikeri determination.

Discussion

Our data indicate that host gene expression does not differ dramatically between uncomplicated symptomatic infections caused by *P. falciparum* or *P. ovale.* This finding is somewhat surprising because species-specific immune responses during *Plasmodium* infections have been described in rodent(44,45) and human(46–48) studies. One possible explanation for this discrepancy is that previous studies compared infections with different severities and/or different infected individuals. The large differences in gene expression observed between infections caused by distinct *Plasmodium* species might therefore have been confounded by host gene expression differences associated with different disease presentations (e.g., severity or symptoms) and/or inter-individual variations. By contrast, our study compared the host gene expression profiles during similar uncomplicated symptomatic infections and in the same individuals, and clearly shows that host factors contribute more to the host gene expression profiles during malaria infections than the infecting *Plasmodium* species. This result is consistent with reports of important interindividual differences(49) in the susceptibility to(50–52) and immune response against(53,54) *P. falciparum* infections.

We observed that the age of the individual significantly contributed to the observed differences in host gene expression. This observation could reflect the maturation of the immune system in young children(55), although, given our small sample size of this study, it is difficult to rigorously evaluate the individual contribution of different host factors (e.g., age, sex or ethnicity) which are confounded in this study. Future studies including more

samples are needed to fully disentangle the role of these host factors and other clinical variables in determining the profiles of the host gene expression during malaria infections. Despite the overwhelming importance of individual factors on the host gene expression, we detected statistically significant differences associated with the infecting species for a small number of human genes, possibly reflecting differences in the host response to these two *Plasmodium* species. We notably found that genes involved in T-cell suppression were highly expressed in *P. falciparum* infections, compared to *P. ovale* infections, consistent with immunological studies showing suppressed T-cell activation during *P. falciparum* infection(56). By contrast, we noted a higher expression of genes related to dendritic cell development during *P. ovale* infections. While the specific role of dendritic cells in *P. ovale* infections has not been studied, nor have the specific innate or adaptive immune responses to this parasite, this observation suggests potential species-specific immune responses and warrants further investigations.

In contrast to the human gene expression results, we found that parasite gene expression differs significantly by species. This could be due to inherent differences in disease features (e.g., parasitemia) or due to true species-specific differences in blood-stage parasite regulation. Several studies have described species-specific gene expression between different species of *Plasmodium* parasites(57,58) but have largely examined species-specific genes(58) and proteins(57). While expression of species-specific variant surface antigens(59) and invasion machinery(60) has been documented, particularly for parasites such as *P. falciparum*(61) and *P. vivax*(62), our data suggest that there may also be species-specific expression of genes present in both genomes (i.e., orthologous genes), including at genes involved in gametocytogenesis or immune modulation.

The RNA-seq data generated also enabled a first glance at the genetic diversity of *P. ovale* in Mali using DNA polymorphisms present in expressed transcripts. Despite the small sample size (only four *P. ovale* infections analyzed), our study revealed the presence of both sub-species of *P. ovale.* Interestingly, both the parasite and host gene expression profiles of infections caused by these highly divergent parasites were very similar compared to the profiles of *P. falciparum* infections. Indeed, we observed more variations among the parasite gene expression profiles of *P. falciparum* infections than between those of *P. ovale curtisi* and *P. ovale wallikeri* infections. In addition, we observed comparable levels of genetic diversity among *P. falciparum* parasites as among *P. ovale curtisi* parasites. This is surprising given the stark difference in prevalence between the two species (and therefore in their population size). While it is difficult to precisely determine the prevalence of *P. ovale,* due to under-detection and species misidentification, *P. ovale* has been reported at about 2% prevalence in Mali compared to ~50% for *P. falciparum*(63). The observation of similar genetic diversity despite drastic differences in (census) population size is puzzling and suggests that i) the prevalence of *P. ovale* in Mali is widely underestimated, due to misdiagnosis or high proportion of asymptomatic infections, or ii) that there is a large hidden reservoir of *P. ovale* parasites. This observation will require validation using larger cohorts but is important to consider as we move closer towards malaria elimination, as it may indicate that some parasite populations are able to maintain a high level of genetic diversity despite little circulation in the population.

Conclusions

Here, we described the transcriptional profiles of host and parasites during malaria infections caused by *P. ovale* or *P. falciparum*. We found that host factors contribute more to the human gene expression profiles than the species causing the infection, suggesting i) that age, sex or other individual host characteristics play a key role in determining the regulation of white blood cells during malaria infections, and ii) that the host responses to *P. ovale* and *P. falciparum* infections are not drastically different (for uncomplicated symptomatic infections). Despite this overall similarity in response, we detected a few human genes differentially regulated in infections with *P. ovale* vs *P. falciparum* suggesting that the host adaptive immune response to these parasites may differ. In addition to insights on the transcriptional regulation of the parasites, this study enabled rigorous characterization of DNA polymorphisms, which revealed the presence of both sub-species of *P. ovale* and a surprisingly high level of genetic diversity in *P. ovale* (comparable to that of *P. falciparum*). Overall, this study provides new insights on the regulation and diversity of *P. ovale* infections that have important implications for the development of pan-malaria vaccines and for developing approaches to eliminate malaria.

Methods

Ethics approval and consent: Individual informed consent/assent was collected from all children and their parents. The study protocol and consent/assent process were approved by the institutional review boards of the Faculty of Medicine, Pharmacy and Dentistry of the University of Maryland, Baltimore and of the University of Sciences,

Techniques and Technologies of Bamako, Mali (IRB numbers HCR-HP-00041382 and HP-00085882).

Samples: Whole-blood samples of Malian children experiencing an uncomplicated symptomatic malaria episode were collected and preserved in PAXgene blood RNA tubes(64). We selected, for these analyses, blood samples from three children successively infected with *P. falciparum* and *P. ovale* (for a total of nine blood samples). The presence of parasites of either species was initially determined by light microscopy using thick blood smears(64). All infections were successfully treated with antimalarial drugs.

Generation of RNA-seq data: We extracted RNA from whole blood using MagMax blood RNA kits (Themo Fisher). Total RNA was subjected to rRNA depletion and polyA selection (NEB) before preparation of stranded libraries using the NEBNext Ultra II Directional RNA Library Prep Kit (NEB). cDNA libraries were sequenced on an Illumina NovaSeq 6000 to generate ~55-130 million paired-end reads of 75 bp per sample (**Supplemental Table 2**). To confirm the *Plasmodium* species responsible for the malaria episode, we first aligned all reads from each sample using hisat2 v2.1.0(65) to a fasta file containing the genomes of *P. falciparum* 3D7, *P. vivax* PvP01, *P. malariae* UG01, and *P. ovale curtisi* GH01 genomes downloaded from PlasmoDB(66) v55. For the remaining analyses described in this study, we relied on the alignment of all reads using hisat2 to a fasta file containing the *P. falciparum* 3D7, *P. ovale* GH01 and human hg38 genomes i) using default parameters and ii) using (--max-intronlen 5000). Reads mapping uniquely

to the hg38 genome were selected from the BAM files generated with the default parameters. Reads mapping uniquely to either *Plasmodium* genome were selected from the BAM files generated with a maximum intron length of 5,000 bp. PCR duplicates were removed from all files using custom scripts. We then calculated read counts per gene using gene annotations downloaded from PlasmoDB (*Plasmodium* genes) and NCBI (human genes) and the subread featureCounts v1.6.4(67).

Gene expression analysis: Read counts per gene were normalized into counts per million (CPM), separately for human and *Plasmodium* genes. To filter out lowly expressed genes, only human genes that were expressed at least at 10 CPM in > 50% of the samples were retained for further analyses (9,884 genes). *Plasmodium* genes were filtered using the same criteria, and additionally selected to only include 1:1 orthologs between *P. falciparum* and *P. ovale* (2,631 genes). Read counts were normalized via TMM for differential expression analyses. Statistical assessment of differential expression was conducted, separately for the human and *Plasmodium* genes, in edgeR (v 3.32.1)(68) using a quasi-likelihood negative binomial generalized model i) without covariates for human genes and ii) with and without correcting for proportion of each parasite developmental stage for *Plasmodium* reads. All results were corrected for multiple testing using false discovery rate (FDR)(69).

Gene expression deconvolution: CIBERSORTx(29) was used to estimate, in each sample, the proportion of i) human immune cell subtypes and ii) *Plasmodium* developmental stages . To deconvolute human reads, we used as a reference LM22(70),

a validated leukocyte gene signature matrix using 547 genes to differentiate 22 immune subtypes (collapsed to eight categories in our analysis). A custom signature matrix derived from *P. berghei* scRNA-seq data was used for *P. falciparum* and *P. ovale* stage deconvolution, using orthologous genes for the appropriate species(37).

Complexity of infection and genotyping: To assess the complexity of each infection (i.e., monoclonal vs. polyclonal), allele frequency plots(71) were generated for each sample by calculating the proportion of reads with a given reference allele at each nucleotide position covered at > 50X. Pairwise nucleotide differences were determined using each position covered at > 20X in a given pair of sample, separately for *P. falciparum* and *P. ovale* infections.

Phylogenetic analysis: We reconstructed the entire *Plasmodium* Cytochrome B sequence from each sample using the mpileup file generated from the RNA-seq data and using, at each nucleotide position covered by at least 20 reads, the allele present in most reads. We then generated a neighbor-joining tree with MEGA 11(72) using the cytochrome B sequences from all sequenced isolates and publicly available sequences for *P. ovale curtisi, P. ovale wallikeri* and *P. vivax* in and 500 bootstraps.

Data availability: All sequence data generated in this study are deposited in the Sequence Read Archive under the BioProject PRJNA878485. Custom scripts are available at https://github.com/tebbenk/PfPo_RNAseq.

Acknowledgements: We thank the participants and their families for participating in this study, as well as the community of Bandiagara, Mali. This work was supported by awards from the National Institutes of Health (R21AI146853 and R01HL146377) and an NIAID-funded predoctoral fellowship (T32 AI095190). Participant enrollment and sample collection were supported by NIH grants U01AI065683, R01HL130750, R01AI099628, and D43TW001589. The funders had no role in study design, data collection and analysis, decision to publish, or preparation of the manuscript.

References

1. World Malaria Report. 2021.
2. Phillips MA, Burrows JN, Manyando C, Van Huijsduijnen RH, Van Voorhis WC, Wells TNC. Malaria. Nat Rev Dis Prim. 2017;3.
3. Milner DA. Malaria pathogenesis. Cold Spring Harb Perspect Med. 2018;8(1):1–11.
4. Berendt AR, Ferguson DJP, Newbold CI. Sequestration in Plasmodium falciparum malaria: Sticky cells and sticky problems. Parasitol Today. 1990;6(8):247–54.
5. Beeson JG, Brown G V. Pathogenesis of Plasmodium falciparum malaria: The roles of parasite adhesion and antigenic variation. Cell Mol Life Sci. 2002;59(2):258–71.
6. Moxon CA, Gibbins MP, McGuinness D, Milner DA, Marti M. New Insights into Malaria Pathogenesis. Annu Rev Pathol Mech Dis. 2020;15:315–43.
7. Brazeau NF, Mitchell CL, Morgan AP, Deutsch-Feldman M, Watson OJ, Thwai KL, et al. The epidemiology of Plasmodium vivax among adults in the Democratic

Republic of the Congo. Nat Commun [Internet]. 2021;12(1):1–10. Available from: http://dx.doi.org/10.1038/s41467-021-24216-3

8. Millar SB, Cox-Singh J. Human infections with Plasmodium knowlesi-zoonotic malaria. Clin Microbiol Infect [Internet]. 2015;21(7):640–8. Available from: http://dx.doi.org/10.1016/j.cmi.2015.03.017

9. Rutledge GG, Böhme U, Sanders M, Reid AJ, Cotton JA, Maiga-Ascofare O, et al. Plasmodium malariae and P. ovale genomes provide insights into malaria parasite evolution. Nature. 2017;542(7639):101–4.

10. Collins WE, Jeffery GM. Plasmodium ovale: Parasite and disease. Clin Microbiol Rev. 2007;20(4):579–92.

11. Collins WE, Jeffery GM. Plasmodium malariae: Parasite and disease. Clin Microbiol Rev. 2007;20(4):579–92.

12. Sutherland CJ, Tanomsing N, Nolder D, Oguike M, Jennison C, Pukrittayakamee S, et al. Two nonrecombining sympatric forms of the human malaria parasite plasmodium ovale occur globally. J Infect Dis. 2010;201(10):1544–50.

13. Oguike MC, Betson M, Burke M, Nolder D, Stothard JR, Kleinschmidt I, et al. Plasmodium ovale curtisi and Plasmodium ovale wallikeri circulate simultaneously in African communities. Int J Parasitol [Internet]. 2011;41(6):677–83. Available from: http://dx.doi.org/10.1016/j.ijpara.2011.01.004

14. Boldt ABW, van Tong H, Grobusch MP, Kalmbach Y, Dzeing Ella A, Kombila M, et al. The blood transcriptome of childhood malaria. EBioMedicine. 2019;40:614–25.

15. Tran TM, Crompton PD, Health G, Biology MI, Section I, Diseases I. Decoding the

complexities of human malaria through systems immunology. 2021;293(1):144–62.

16. Jonathan Posner and Bradley S. Peterson JAR. Systems immunology of human malaria Tuan. Trends Parasitol. 2012;28(6):248–57.

17. Otto TD, Wilinski D, Assefa S, Keane TM, Sarry LR, Böhme U, et al. New insights into the blood-stage transcriptome of Plasmodium falciparum using RNA-Seq. Mol Microbiol. 2010;76(1):12–24.

18. Bozdech Z, Llinás M, Pulliam BL, Wong ED, Zhu J, DeRisi JL. The transcriptome of the intraerythrocytic developmental cycle of Plasmodium falciparum. PLoS Biol. 2003;1(1):85–100.

19. Hoo R, Zhu L, Amaladoss A, Mok S, Natalang O, Lapp SA, et al. Integrated analysis of the Plasmodium species transcriptome. EBioMedicine [Internet]. 2016;7:255–66. Available from: http://dx.doi.org/10.1016/j.ebiom.2016.04.011

20. Sims JS, Militello KT, Sims PA, Patel VP, Kasper JM, Wirth DF. Patterns of gene-specific and total transcriptional activity during the Plasmodium falciparum intraerythrocytic developmental cycle. Eukaryot Cell. 2009;8(3):327–38.

21. Dia A, Jett C, McDew-White M, Li X, Anderson TJC, Cheeseman IH. Efficient transcriptome profiling across the malaria parasite erythrocytic cycle by flow sorting. 2020;

22. Rovira-Graells N, Gupta AP, Planet E, Crowley VM, Mok S, De Pouplana LR, et al. Transcriptional variation in the malaria parasite Plasmodium falciparum. Genome Res. 2012;22(5):925–38.

23. Bradwell KR, Coulibaly D, Koné AK, Laurens MB, Dembélé A, Tolo Y, et al. Host

and Parasite Transcriptomic Changes upon Successive Plasmodium falciparum Infections in Early Childhood . mSystems. 2020;5(4):1–13.

24. Lee HJ, Walther M, Georgiadou A, Nwakanma D, Stewart LB, Levin M, et al. Integrated pathogen load and dual transcriptome analysis of systemic host-pathogen interactions in severe malaria. bioRxiv. 2017;10(447):1–31.

25. Yamagishi J, Natori A, Tolba MEM, Mongan AE, Sugimoto C, Katayama T, et al. Interactive transcriptome analysis of malaria patients and infecting Plasmodium falciparum. Genome Res. 2014;24(9):1433–44.

26. Daily JP, Le Roch KG, Sarr O, Ndiaye O, Lukens A, Zhou Y, et al. In vivo transcriptome of plasmodium falciparum reveals overexpression of transcripts that encode surface proteins. J Infect Dis. 2005;191(7):1196–203.

27. Brashear AM, Roobsoong W, Siddiqui FA, Nguitragool W, Sattabongkot J, López-Uribe MM, et al. A glance of the blood stage transcriptome of a Southeast Asian Plasmodium ovale isolate. PLoS Negl Trop Dis. 2019;13(11):1–20.

28. Hoffman GE, Schadt EE. variancePartition: Interpreting drivers of variation in complex gene expression studies. BMC Bioinformatics [Internet]. 2016;17(1):17–22. Available from: http://dx.doi.org/10.1186/s12859-016-1323-z

29. Newman AM, Steen CB, Liu CL, Gentles AJ, Chaudhuri AA, Scherer F, et al. Determining cell type abundance and expression from bulk tissues with digital cytometry. Nat Biotechnol. 2019;37(7):773–82.

30. Shinde R, Shimoda M, Chaudhary K, Liu H, Mohamed E, Bradley J, et al. B Cell–Intrinsic IDO1 Regulates Humoral Immunity to T Cell–Independent Antigens. J Immunol. 2015;195(5):2374–82.

31. Merlo LMF, DuHadaway JB, Montgomery JD, Peng WD, Murray PJ, Prendergast GC, et al. Differential Roles of IDO1 and IDO2 in T and B Cell Inflammatory Immune Responses. Front Immunol. 2020;11(August):1–16.

32. Hunter CA. New IL-12-family members: IL-23 and IL-27, cytokines with divergent functions. Nat Rev Immunol. 2005;5(7):521–31.

33. Villarino A, Hibbert L, Lieberman L, Wilson E, Mak T, Yoshida H, et al. The IL-27R (WSX-1) is required to suppress T cell hyperactivity during infection. Immunity. 2003;19(5):645–55.

34. Hunter CA, Villarino A, Artis D, Scott P. The role of IL-27 in the development of T-cell responses during parasitic infections. Immunol Rev. 2004;202(2):106–14.

35. Wang LT, Lin MH, Liu KY, Chiou SS, Wang SN, Chai CY, et al. WLS/wntless is essential in controlling dendritic cell homeostasis via a WNT signaling-independent mechanism. Autophagy [Internet]. 2021;17(12):4202–17. Available from: https://doi.org/10.1080/15548627.2021.1907516

36. Zuidscherwoude M, Worah K, Van Der Schaaf A, Buschow SI, Van Spriel AB. Differential expression of tetraspanin superfamily members in dendritic cell subsets. PLoS One. 2017;12(9):1–15.

37. Tebben K, Dia A. Determination of the Stage Composition of Plasmodium Infections from Bulk Gene Expression Data. 2022;XX(Xx).

38. Kehrer J, Singer M, Lemgruber L, Silva PAGC, Frischknecht F, Mair GR. A Putative Small Solute Transporter Is Responsible for the Secretion of G377 and TRAP-Containing Secretory Vesicles during Plasmodium Gamete Egress and Sporozoite Motility. PLoS Pathog. 2016;12(7):1–25.

39. Kumar S, Leeb AS, Vaughan AM, Kappe SHI. Plasmodium falciparum Cysteine Rich Secretory Protein uniquely localizes to one end of male gametes. Mol Biochem Parasitol [Internet]. 2022;248(January):111447. Available from: https://doi.org/10.1016/j.molbiopara.2022.111447

40. Nasamu AS, Polino AJ, Istvan ES, Goldberg DE. Malaria parasite plasmepsins: More than just plain old degradative pepsins. J Biol Chem. 2020;295(25):8425–41.

41. Cui A, Li Y, Zhou X, Wang L, Luo E. Characterization of plasmodium berghei homologues of t-cell immunomodulatory protein as a new potential candidate for protecting against experimental cerebral malaria. Korean J Parasitol. 2019;57(2):101–15.

42. Nabet C, Doumbo S, Jeddi F, Konaté S, Manciulli T, Fofana B, et al. Genetic diversity of Plasmodium falciparum in human malaria cases in Mali. Malar J. 2016;15(1):1–10.

43. 2020 W. World Malaria Report 2020 [Internet]. Vol. 73, Who. 1997. 1–4 p. Available from: https://www.who.int/teams/global-malaria-programme/reports/world-malaria-report-2020

44. Inoue M, Tang J, Miyakoda M, Kaneko O, Yui K, Culleton R. The species specificity of immunity generated by live whole organism immunisation with erythrocytic and pre-erythrocytic stages of rodent malaria parasites and implications for vaccine development. Int J Parasitol [Internet]. 2012;42(9):859–70. Available from: http://dx.doi.org/10.1016/j.ijpara.2012.07.001

45. Voza T, Vigário AM, Belnoue E, Grüner AC, Deschemin JC, Kayibanda M, et al.

Species-specific inhibition of cerebral malaria in mice coinfected with Plasmodium spp. Infect Immun. 2005;73(8):4777–86.

46. Andrysiak PM, Collins WE, Campbell GH. Stage-specific and species-specific antigens of Plasmodium vivax and Plasmodium ovale defined by monoclonal antibodies. Infect Immun. 1986;54(3):609–12.

47. Taylor DW, Jin Kim K, Munoz PA, Evans CB, Asofsky R. Monoclonal antibodies to stage-specific, species-specific, and cross-reactive antigens of the rodent malarial parasite, Plasmodium yoelii. Infect Immun. 1981;32(2):563–70.

48. Burel JG, Apte SH, McCarthy JS, Doolan DL. Plasmodium vivax but Not Plasmodium falciparum Blood-Stage Infection in Humans Is Associated with the Expansion of a CD8+T Cell Population with Cytotoxic Potential. PLoS Negl Trop Dis. 2016;10(12):1–11.

49. Gupta S, Hill AVS. Dynamic interactions in malaria: Host heterogeneity meets parasite polymorphism. Proc R Soc B Biol Sci. 1995;261(1362):271–7.

50. Driss A, Hibbert JM, Wilson NO, Iqbal SA, Adamkiewicz T V., Stiles JK. Genetic polymorphisms linked to susceptibility to malaria. Malar J [Internet]. 2011;10(1):271. Available from: http://www.malariajournal.com/content/10/1/271

51. Mockenhaupt FP, Cramer JP, Hamann L, Stegemann MS, Eckert J, Oh NR, et al. Toll-like receptor (TLR) polymorphisms in African children: Common TLR-4 variants predispose to severe malaria. Proc Natl Acad Sci U S A. 2006;103(1):177–82.

52. Recker M, Valletta JJ, Addy JWG, Reid AJ, Ndungu FM, Bediako Y, et al. Individual-level variations in malaria susceptibility and acquisition of clinical

protection. Wellcome Open Res. 2022;6.

53. Korbel DS, Newman KC, Almeida CR, Davis DM, Riley EM. Heterogeneous Human NK Cell Responses to Plasmodium falciparum -Infected Erythrocytes . J Immunol. 2005;175(11):7466–73.

54. Yap XZ, McCall MBB, Sauerwein RW. Fast and fierce versus slow and smooth: Heterogeneity in immune responses to Plasmodium in the controlled human malaria infection model. Immunol Rev. 2020;293(1):253–69.

55. Simon AK, Hollander GA, McMichael A. Evolution of the immune system in humans from infancy to old age. Proc R Soc B Biol Sci. 2015;282(1821).

56. Frimpong A, Kusi KA, Adu-Gyasi D, Amponsah J, Ofori MF, Ndifon W. Phenotypic evidence of T cell exhaustion and senescence during symptomatic plasmodium falciparum Malaria. Front Immunol. 2019;10(JUN):1–11.

57. Hillier C, Pardo M, Yu L, Bushell E, Sanderson T, Metcalf T, et al. Landscape of the Plasmodium Interactome Reveals Both Conserved and Species-Specific Functionality. Cell Rep. 2019;28(6):1635-1647.e5.

58. Sa JM, Cannon M V., Caleon RL, Wellems TE, Serre D. Single-cell transcription analysis of Plasmodium vivax blood-stage parasites identifies stage- and species-specific profiles of expression [Internet]. Baltimore, MD; 2020. Available from: https://journals.plos.org/plosbiology/article?id=10.1371/journal.pbio.3000711

59. Langhorne J, Ndungu FM, Sponaas AM, Marsh K. Immunity to malaria: More questions than answers. Nat Immunol. 2008;9(7):725–32.

60. Cowman AF, Tonkin CJ, Tham WH, Duraisingh MT. The Molecular Basis of Erythrocyte Invasion by Malaria Parasites. Cell Host Microbe [Internet].

2017;22(2):232–45. Available from: http://dx.doi.org/10.1016/j.chom.2017.07.003

61. Hayton K, Gaur D, Liu A, Takahashi J, Henschen B, Singh S, et al. Erythrocyte Binding Protein PfRH5 Polymorphisms Determine Species-Specific Pathways of Plasmodium falciparum Invasion. Cell Host Microbe. 2008;4(1):40–51.

62. Batchelor JD, Malpede BM, Omattage NS, DeKoster GT, Henzler-Wildman KA, Tolia NH. Red Blood Cell Invasion by Plasmodium vivax: Structural Basis for DBP Engagement of DARC. PLoS Pathog. 2014;10(1).

63. Traore K, Thera MA, Bienvenu AL, Arama C, Bonnot G, Lavoignat A, et al. Interaction between environment, nutrient-derived metabolites and immunity: A possible role in malaria susceptibility/resistance in fulani and dogon of Mali. PLoS One. 2017;12(12):1–15.

64. Coulibaly D, Travassos MA, Kone AK, Tolo Y, Laurens MB, Traore K, et al. Stable malaria incidence despite scaling up control strategies in a malaria vaccine-testing site in Mali. Malar J. 2014;13(1):1–9.

65. Kim D, Paggi JM, Park C, Bennet C, Salzberg SL. Graph-based genome alignment and genotyping with HISAT2 and HISAT-genotype. Nat Biotechnol [Internet]. 2019;37:907–15. Available from: https://www.nature.com/articles/s41587-019-0201-4

66. Aurrecoechea C, Brestelli J, Brunk BP, Dommer J, Fischer S, Gajria B, et al. PlasmoDB: A functional genomic database for malaria parasites. Nucleic Acids Res. 2009;37(SUPPL. 1):539–43.

67. Liao Y, Smyth GK, Shi W. FeatureCounts: An efficient general purpose program for assigning sequence reads to genomic features. Bioinformatics.

2014;30(7):923–30.

68. Robinson MD, McCarthy DJ, Smyth GK. edgeR: A Bioconductor package for differential expression analysis of digital gene expression data. Bioinformatics. 2009;26(1):139–40.

69. Benjamini Y, Hochberg Y. Controlling the False Discovery Rate : A Practical and Powerful Approach to Multiple Testing Author (s): Yoav Benjamini and Yosef Hochberg Source : Journal of the Royal Statistical Society . Series B (Methodological), Vol . 57 , No . 1 (1995), Publi. J R Stat Soc. 1995;57(1):289–300.

70. Newman AM, Liu CL, Green MR, Gentles AJ, Feng W, Xu Y, et al. Robust enumeration of cell subsets from tissue expression profiles. Nat Methods. 2015;12(5):453–7.

71. Chan ER, Menard D, David PH, Ratsimbasoa A, Kim S, Chim P, et al. Whole Genome Sequencing of Field Isolates Provides Robust Characterization of Genetic Diversity in Plasmodium vivax. PLoS Negl Trop Dis. 2012;6(9).

72. Tamura K, Stecher G, Kumar S. MEGA11: Molecular Evolutionary Genetics Analysis Version 11. Mol Biol Evol. 2021;38(7):3022–7.

Supplemental Table 1

Click here to access/download
Supporting Information
supplemental_table1.xlsx

Supplemental Figures 1-5 and legends

Click here to access/download
Supporting Information
supporting_information.docx

Supplemental table 2

Click here to access/download
Supporting Information
supplemental_table2.xlsx

Supplemental table 3

Click here to access/download
Supporting Information
supplemental_table_3.xlsx

Supplemental table 4

Click here to access/download
Supporting Information
supplemental_table_4.xlsx
