# Supplementary material for: Malian children infected with Plasmodium ovale and Plasmodium falciparum display very similar gene expression profiles
Source: PLoS Negl Trop Dis. 2023 Jan 25;17(1):e0010802. doi: 10.1371/journal.pntd.0010802 (PMC9901758; doi:10.1371/journal.pntd.0010802)
Supplement: Attachment — Submitted filename: ResponseNTD_final.docx [file pntd.0010802.s011.docx]

We would like to thank the reviewers for their detailed review of our manuscript and their constructive comments and suggestions. We have now extensively modified the manuscript according to their suggestions and present below a detailed response to their comments (the page/line numbers refer to the manuscript without track changes).

**Editor’s comments**

You will noticed that that the reviewers are persuaded of the importance of your study. However, they have raised several points that require consideration and revision. One reviewer has noted that there is no replication of the RNAseq analysis. I view this as a substantive concern. Replication is important as the estimation of P values depends on the between-replicate variance, and replication is good scientific practise in any experiment. As these P values underpin a substantial component of this manuscript, a requirement for resubmission is that the RNAseq analysis is replicated.

We have addressed in details the reviewers’ comments below. To briefly summarize the discussion regarding the need of “replication”: RNA-seq has been extensively validated since its introduction, 15 years ago, and technical validation by RT-PCR is widely considered unnecessary and often uninformative (since the sensitivity of qRT-PCR is typically much lower than that of RNA-seq, one would require a very large number of replicates to have sufficient power to validate many of the gene expression differences identified by RNA-seq). We fully agree that biological replication, using additional samples, would improve this study but, unfortunately, this is currently not feasible due to the difficulty to obtain samples infected with *P. ovale*. We have added substantial discussion in our manuscript describing these limitations and the preliminary nature of our findings. However, we would also like to emphasize that many RNA-seq studies published from non-falciparum infections have similar, or lower, sample size, reflecting the difficulties to study these parasites (see below for details).

**Reviewer #1**:

In the manuscript by Tebben et al, the authors characterized host and parasite gene expression profiles during Plasmodium ovale versus Plasmodium falciparum infections to dissect molecular mechanisms at play during malaria infections by different Plasmodium parasites. Accordingly, most studies focus on P. falciparum, other Plasmodium species also cause significant public health issues and there is a lack of specific knowledge about these parasites. Additionally, other Plasmodium species, such as P. ovale, typically lead to less severe forms of the disease but little is known about the molecular mechanisms of those non-falciparum infections. In this manuscript, the authors attempted a rare approach of sequencing both host and parasite RNA from nine children infected with either P. falciparum or P. ovale parasites.

They found that in uncomplicated symptomatic infections, host gene expression profiles differ dramatically according to the participant age rather than by the infecting parasite species. However, parasite gene expression profiles differ between Plasmodium species. They also show that the levels of parasite genetic diversity analyzed using DNA polymorphisms from RNA-seq reads were comparable between Plasmodium falciparum and ovale, despite a dramatic difference in prevalence.

Although the number of analysed samples is modest, the manuscript is informative and well presented, and particularly interesting for the understudied P. ovale species. Nonetheless, some precisions and analyses are required before publication.

Thank you for your kind assessment of our study.

Comments:

-	More epidemiological data is needed on the collected blood samples. Including: symptoms in each patient, date of sample collection (to infer time in between subsequent infections) and season (wet/dry) of sample collection. In Table 1, some individuals have a body temperature < 37.5°C. What was the definition of ‘uncomplicated malaria’ ? Were patients tested for other co-infections (such as helminth)? Sample B3 is not feverish and parasitaemia is more typical of an asymptomatic infection. Why is it classified as uncomplicated malaria?

We have now added more details have in Table 1 and on page 21 of the Materials and Methods. We are unfortunately limited by the epidemiological data obtained by the study team at the time of collection of these samples (Coulibaly et al. Malaria Journal 2014). The data presented in Table 1 now include the Month of collection, the season (dry/wet), the parasitemia, body temperature and hemoglobin concentration.

All blood samples analyzed in this study were collected from an unscheduled visit, initiated by the patient in response to (self-assessed) malaria symptoms, with microscopic evidence of parasites. We followed in our study the definition of ‘clinical malaria’ used by the study clinicians and defined by “treatment seeking behavior, clinical symptoms consistent with malaria (fever, headaches, joint pain, vomiting, diarrhea, or abdominal pain) and presence of malaria parasites at any density” (Coulibaly et al. Malaria Journal 2014). This clinical definition of malaria resulted in the administration of antimalarial medication to the patient. We do not have documentation of the specific symptoms that each child presented with at the time of collection. However, we agree with the reviewer’s concern and have removed the “symptomatic” terminology from the manuscript since it is possibly problematic and did not add value to the study.

We appreciate the reviewer’s concern about the possibility of co-infections such as helminths. Previous work has demonstrated a significant burden of schistosomiasis in the community, but failed to detect filarial infections (Lyke et al., AJTMH 2005). The routine capacity to diagnose such co-infections was not available in the field (reflecting the limited capacity for such testing at a remote rural sub-Saharan clinic). Note that analyses of the RNA-seq data did not reveal evidence of transcripts from other organisms (e.g., no microfilaria RNAs were detected).

-	Line 410-413: Only reads mapping to the corresponding target genome (hg38, P. falciparum P. ovale) were selected. What is the proportion of reads mapping to both falciparum and ovale why do you exclude them? Unless I misunderstand the methods, it sounds like reads that map to both falciparum and ovale are discarded.

Given the read length used, very few reads map equally well to the *P. falciparum* and *P. ovale* genome (typically less than 1%, see e.g., Supplemental Table 1). The reads mapping equally well to two genomes often derive from ribosomal RNA genes that are very conserved across *Plasmodium* species but are uninformative for our analyses and we therefore excluded these reads from subsequent analyses.

-	In general, Methods should be more detailed. Were samples frozen before RNA extraction? What RNA quantity was recovered in each sample?

We have now expanded the methods on page 21. All PAXgene tubes were stored at -80C upon collection and until RNA extraction. 0.24 – 3.16 ug of RNA was extracted from each sample.

-	The lack of replicates is a limitation of the study, this should be clearly stated.

The limitations of the study, including the lack of replicates and the small sample size, are now explicitly discussed throughout the manuscript (e.g., lines 137-140, 268-270, 298, 347-349, 362-363, 401-404).

-	Line 431: Gene expression deconvolution. The rodent P. berghei scRNAseq data was used to determine the developmental age of each sample. That species does not sequester and, unlike falciparum, all stages are circulating in the blood. As mentioned on line 232, there is a risk that the stage composition is biased. In my experience, the method developed by Lemieux et al (2009 PNAS, Newbold lab) is more accurate, I’m happy to share a R script to determine the parasite developmental age based on the microarray timecourse (data Bozdech 2003). Also, are blood smears available? What was the proportion of ring/troph/gametocytes on these blood smears?

The comparison of falciparum versus ovale transcriptomes (Fig 2) only makes sense if all samples are at the exact same developmental stage (if not, a normalizing step should be added , similar to Thomson-Luque2021 Nature Communications, Portugal lab)

As noted by the reviewer, estimating the parasite developmental age is a powerful method of correcting for stage differences. However, this approach works best when the parasite population of one sample is relatively homogenous (and unimodal) and only differ from the parasite population of another sample by its mean age (as is typically the case for synchronized cultures of *P. falciparum* for which this method was developed). By contrast, parasites characterized by bulk RNA-seq data directly from patient blood (i.e., without culture) often display complex stage heterogeneity (and a multimodal stage distribution) that is better corrected for by using estimates of the different stage proportions. This stage heterogeneity is supported by three main ideas. First, it is unclear whether sequestration occurs in *P. ovale* (or to what extent) and many asexual stages might be concurrently present in the circulation. Second, even when there is sequestration of mature stages, the transcriptional profiles might be more heterogeneous that expected based on microscopy since rings typically display a lower transcriptional activity than other stages and therefore contribute little to the overall gene expression profiles despite their abundance. Third and more importantly, gametocytes are not accounted for using developmental age, which can introduce important biases in unprocessed patient samples. These important points and a general discussion of the limitations of these methods is now presented on page 18.

Unfortunately, no blood smears are available for these samples, preventing comparisons of the microscopy and gene expression deconvolution results (although see Kim et al., Nature Communications 2019 for such comparisons and a more thorough discussion). Difference in stage compositions between *P. ovale* and *P. falciparum* samples is discussed on lines 247-257 (page 12).

-	Line 439: Complexity of infection. Supp Fig 4 is not very convincing that all samples are monoclonal. I recommend calculating the Fws metric on all samples (Auburn 2012 Plos One)

We have now included estimates of Fws for all samples on Supplemental Figure 4 and discuss these findings in the text on page 14.

-	There is no coexpression analysis combining both datasets (host & parasite) together, as in Lee et al 2018 Science Trans Med. The findings from that paper (human differential gene expression driven by parasite load) should be discussed with the results presented here.

We do not feel that the sample size of this study would support robust co-expression analysis, especially with regards to the parasite load that is (partially) confounded with the *Plasmodium* species (see also below).

Side note: in the References section, Lee 2018 is cited on BioRxiv rather than the final publication.

We have now fixed the reference. Thank you for noticing this.

- In most figures, only 8 samples are presented instead of the 9 listed in Table 1.

We excluded one sample (B4) from all analyses due to its very high proportion of PCR duplicates (~95%) reflecting the poor quality of the mRNAs. This is now clearly stated on page 6 (lines 122-124) and page 22.

-	Line 175-182. Among the 127 host DE genes, are genes associated with inflammation and what is the direction of regulation (up or down-regulated) according to the two plasmodial species?

Given the small sample size, we chose to focus our analyses and discussion on qualitative differences in gene expression and comparison of the number of differentially expressed genes between similar groups rather than on the actual genes. However, in response to the reviewer’s comment, we have now expanded the discussion of inflammation-related genes on pages 9 and 17 (and highlighted that these results should be interpreted with caution). Briefly, a few genes related to innate inflammation (inflammasome activation, antigen presentation, complement system) showed increased expression in *P. ovale* infections, while some genes related to dendritic cell activation/development were more highly expressed in *P. falciparum* infections.

-	Line 273: delete "(" at the beginning of the sentence

Done.

-	Supplementary Figure 3 is unclear and is not mentioned in the main text.

Supplemental Figure 3 is discussed on line 245 and we have expanded its legend.

-	I probably did something wrong, but I couldn’t retrieve the Bioproject ‘PRJNA878485’ ?

This submission was under embargo until publication. We have now changed the permissions and the data should be freely accessible.

**Reviewer #2:**

This study is quite innovative, trying to compare the blood transcriptome (of host and parasite) between P falciparum and P ovale infections. P ovale is relatively neglected in terms of research, and difficult to study because it is much less common than P falciparum and often exists as co-infections. Therefore the comparison of P falciparum and P ovale monoinfections in this way is novel and the authors have undertaken quite extensive analysis to investigate both host and parasite biology. Studying these during sequential mono-infections in the same subjects is a novel approach but unfortunately it is very hard to collect such samples and so only 3 subjects are included in the study.

My biggest concern is that the subjects are described as having "symptomatic malaria" but the evidence presented in Table 1 does not strongly support this. Several of the infection episodes are associated with lack of fever, and in some cases the parasitemias are very low. No mention is made of the case definition of "symptomatic malaria" and there is no report of what other microbiological, virological, and parasitological investigations were done to rule out alternative sources of fever, which will be common in children of this age in Mali. The onus is on the authors to convince us that these children really did all have symptomatic malaria, without co-infections, and that parasitemia was not simply an incidental finding during some of these episodes. This will potentially have a major impact on the interpretation of all subsequent analysis of the host response and possibly the parasite transcriptome

We have updated the main text and the methods sections to better describe the infections and have removed all references to symptomatic infections (see response to Reviewer 1 for details).

My next major concern is that the sample size is extremely small.

We do not disagree with the reviewer’s comment on the limited sample size but would like to emphasize the difficulty to collect and analyze non-falciparum infections, as she/he noted. Indeed, several studies of non-falciparum *Plasmodium* infections have been published with roughly similar small number: Zhu et al., Sci. Rep 2016 (n=3), Brashear et al., PLoS NTD 2019 (n=1), Kim et al., Sci. Rep 2017 (n=3), Gunalan et al., PNAS 2019 (n=2 vs. 2). This does not alleviate the concerns of the reviewer but highlights the difficulties to collect and analyze such samples. We have more extensively discussed this limitation throughout the manuscript (see response to Reviewer 1 for details).

Presumably this small sample size precludes adjustment for leukocyte mixture in the analysis of host gene expression? Nevertheless 127 differentially expressed host genes were found between P ovale and P falciparum infections. The authors state that this confirms the overall similarity of the host response to these infections. However I consider it quite remarkable that with this small number of subjects it was possible to detect so many differentially expressed genes, and that with larger sample sizes there could easily be a much larger number of differentially expressed genes. This can potentially be resolved by performing a formal power calculation to understand the proportion of differentially expressed genes which might be detected with a sample size of just 3 subjects.

We agree that inclusion of additional samples would likely yield a greater number of differentially expressed genes. However, our initial statement derived from the comparison of the host and parasite gene expression profiles (that are based on the same sample size): while 1,858 parasite genes were differentially expressed between Po and Pf infections, “only” 127 host genes were differentially expressed (based on identical sample size and statistical cutoff). We have now clarified this statement (on page 8 and 11) and explicitly discussed the limitations of the small sample size.

Similarly the authors suggest that the differential expression is not due to differences in leukocyte proportions in blood, based on Chi-squared test P>0.1. Again they should perform a power calculation to determine what magnitude of difference in proportions of cell types they would be able to detect at a significance threshold of P<0.1 (or P<0.05). I suspect they would only be able to detect extremely large differences with confidence.

We apologize for the poor wording of this section: our analysis showed no evidence of differences in WBC subset compositions (consistent with the relatively small number of host genes differentially expressed) but does not indicate that these cell compositions are identical. We agree with the limited power of this analysis, although simulations indicate that a 10% increase can be rigorously detected under specific conditions. We have now edited this section on page 8.

The approach of showing the variance in gene expression explained by species, age, parasitemia and individual is quite nice, but as the authors acknowledge out, age and individual are are not independent, and no account has been taken of the fact that parasitemia and parasite species are also not independent of one another - typically P vivax infections have lower parasitemia than P falciparum. Therefore this analysis is unfortunately flawed, and would need to be modified to account for this.

The reviewer brings out a valid criticism and this limitation is now clearly stated on page 17, although it is worth noting that the parasitemia and species are not entirely confounded (one Pf infection has the lowest parasitemia, one Po and one Pf infections have comparable parasitemia). In addition, association of gene expression with the species is categorical (binary) while the association with parasitemia is quantitative (or monotonic) and it is therefore possible for the expression of one gene to be statistically associated with the *Plasmodium* species but not the parasitemia.

The use of orthologous genes to estimate parasite developmental stage in analysis of parasite gene expression is good, but the authors should mention that the stage specific gene expression of P ovale is not as well known as P falciparum, so it may not be as accurate.

The reviewer is correct and this is now clearly stated on page 12/13. Note that the deconvolution has been tested on highly divergent *Plasmodium* species (Tebben et al., 2022) and that, since *P. ovale* falls within this range, we do not have any reason to expect its stage specific expression to differ dramatically (but we do not have any actual data to confirm or invalidate this hypothesis).

Similar to my comment above about the human gene expression, the interpretation of parasite gene expression also needs to be tempered by the statistical power of the analysis: "Accounting for stage composition differences, only 118 orthologous genes remained differentially expressed between parasite species". 118 differentially expressed genes actually sounds like a lot for such a small sample size.

This statement was meant in comparison to the ~1800 genes differentially expressed when the analyses were performed without accounting for stage composition. But the reviewer is correct and we have now moderated and clarified this statement on page 13.

In light of these comments there is a need to undertake some substantial reanalysis and revision of the title, results and discussion text accordingly.

We have now extensively edited (and moderated) our statements according to the reviewers’ comments.

Minor comment:

Gene symbols need to be corrected to standard nomenclature for human genes ie all Upper Case.

Changed. Thank you for noticing this.

**Reviewer #3:**

Could the authors mention about the time-point of collection?

The collection times of each sample are now included in Table 1. Thank you for the suggestion.

Were the sequencing depth of approximately 55 - 130 million paired-end reads sufficient to obtain sequence coverage of either transcriptome?

This sequencing depth is greater than recommended for human studies (~30 million reads) and similar to other studies of *Plasmodium* infections (see table below for examples).

| **Study** | **Number of reads** |
| --- | --- |
| Zhu et al., 2016 (Sci. Reports) | *P. vivax*: 4,400,000 to 10,200,000 |
| Brashear et al., 2017 (PlosNTD) | *P. ovale:* 11,381,290 |
| Lee et al., 2018 (Sci Trans Med) | Human: median 26,600,000  *P. falciparum*: median 5,030,000 (uncomplicated infections) |
| Bradwell et al., 2020 (mSystems) | Total: 34,000,000 to 67,000,000  Human: 5,000,000 to 24,000,000  *P. falciparum:* 1,000,000 to 12,000,000 |
| Kim et al., 2017 (Sci Reports) | Human: avg. 28,974,130  *P. vivax*: avg. 6,618,837 |

Was it necessary to consider a validation of the transcriptome by RT-qPCR of the phenotype of interest?

RNA-seq is a well-validated approach and there is a general consensus that technical validation by qRT-PCR is unnecessary and often unfeasible: given the difference in sensitivity of the two methods, qPCR validation of genes differentially expressed by even 1.5-fold would require a very large number of replicates. Biological replicates would be great, but we unfortunately do not have access to more infections (see also response to Reviewer 2). Note also that our main findings (Figs 1, 2 and 3) rely on the overall number of differentially expressed genes (and their relative changes between comparisons) rather than on the p-value of a single gene.

What were the statistical considerations when analyzing the uneven replicate data set of your three subjects?

As noted in the response to Reviewer 1, sample B4 was excluded from all analyses due to the high proportion of PCR duplicate reads (now clarified on page 5 and the legend of Table 1).

Since the variance analysis (Fig 1) showed that human gene expression profiles were primarily influenced by the individual/age, we chose to use a paired design to test how the host gene expression differed between Po and Pf infections: we selected the closest (in time) Pf and Po infections from each individual and tested these three pairs.

For the parasite gene expression, the variance analysis (Fig 2) supported an overwhelming effect of the parasite species and we therefore decided to include all 8 samples (four Po infections and four Pf infections) in a (non-paired) analysis. However, based on the reviewer’s comment we have no added a Supplemental Figure (S2D) presenting the parasite gene expression analysis using the exact same samples as used in the host gene expression and using the same paired design.

We have more clearly noted this in the text on pages 8 and 11-12 and in the figure legend of Supplemental Figures 1 and 2.

Supplemental Figure 2 panel C, the legend needs editing

Fixed
